# Things left unsaid: important topics that are not discussed between patients with systemic sclerosis, their carers and their healthcare professionals—a discourse analysis

**DOI:** 10.1007/s10067-020-05371-2

**Published:** 2020-09-11

**Authors:** Christopher P. Denton, Bee Laird, Lizette Moros, Jose Luis Luna Flores

**Affiliations:** 1 Division of Medicine, Department of Inflammation, Centre for Rheumatology and Connective Tissue Diseases, Royal Free and University College Medical School, University College London, London, UK; 2 The Research Unit, Hove, UK; 3 Boehringer Ingelheim International GmbH, Ingelheim am Rhein, Germany

**Keywords:** Interstitial lung disease, Interstitial lung fibrosis, Patient–physician communication, Specialist nurse, Systemic sclerosis

## Abstract

**Introduction:**

Systemic sclerosis (SSc) is a rare condition that can be complicated by interstitial lung fibrosis (SSc-ILD)—a major cause of mortality. This study explored information and communication needs of patients with SSc-ILD and their carers to understand what they are and whether they are met.

**Methods:**

Qualitative research was performed, including in-depth individual interviews and observed conversations between pairs of patients, physicians and nurses, and between patients and physicians discussing experiences of SSc-ILD. The study was performed in Germany, Italy, Spain, the UK and the USA. Participants included 42 SSc-treating physicians, 21 patients with diagnosed SSc-ILD, 16 specialist nurses and five carers.

**Results:**

Prognosis and mortality were the main unspoken topics acknowledged by patients, carers and healthcare professionals. Patients and carers felt afraid to ask physicians about mortality, and most physicians reported avoiding the question because their duty was to give patients hope and avoid causing additional distress. Patients often felt unable to ask physicians about relationships, family and work because of time constraints or because they felt these were not topics physicians would be concerned about. Often, specialist nurses felt that they had insufficient knowledge to provide adequate support.

**Conclusion:**

Key topics, including mortality and prognosis, are rarely openly discussed, leaving patients uncertain and anxious about the future. By communicating about difficult but important topics, physicians and nurses could help patients and carers manage and plan their lives. This study shows that a multi-professional team-based communication approach is likely to better address patient needs and priorities.

****Key Points**:**

• *Key topics in SSc or SSc-ILD, such as mortality and prognosis, are rarely openly discussed in clinical consultations.*

• *By communicating difficult but important topics, physicians and nurses could help patients manage their disease and plan their lives.*

• *A multi-professional team-based communication approach is likely to better address patient needs and priorities and could be easily implemented without the need for significant additional resources.*

## Introduction

Systemic sclerosis (SSc) is a rare, serious and complex disease, characterised by progressive organ damage, including the development of interstitial lung disease (ILD) and neurological, cardiovascular, renal and digestive tract dysfunction [1–3]. The variability and unpredictability of disease progression are a significant cause of anxiety, with many patients fearful of what their future holds [4]. SSc in all its manifestations, including SSc associated with ILD (SSc-ILD), substantially affects a person’s health, general wellbeing and quality of life [5], as well as reducing life expectancy [6, 7].

Consultations between patients with SSc and healthcare professionals (HCPs) often follow different patterns, depending on the individual’s journey and their circumstances. In addition, different assumptions may be made by patients and their healthcare providers with respect to the other’s knowledge and understanding of disease pathology, associated physical limitations, psychological impact, prognosis and life expectancy [8].

SSc is dynamic, and patients constantly need to adapt to their changing (dis)abilities and adopt new coping strategies [9]. Guidance and support from HCPs regarding adaptations and disease management would therefore be welcome.

Patients with SSc-ILD may have additional concerns related to their lung involvement. ILD is the biggest cause of death in SSc [10], and this is reported in much of the currently available online patient information. It is not clear from the existing research how much the increased risk of death impacts on patients’ needs for information and support, whether they have specific needs in relation to SSc-ILD-associated mortality and whether these concerns are openly discussed in patients’ interactions with their physicians.

We aimed to explore and identify information and communication needs of patients with SSc-ILD and their caregivers, specifically highlighting topics that are typically not discussed in consultations with physicians, and to establish the reasons these topics are not addressed.

## Methods

### Observations

This qualitative study was conducted in Germany, Italy, Spain, the United Kingdom (UK), and the United States of America (USA). To enable critical discourse analysis, the study captured the natural language from three different types of interactions with patients with SSc-ILD, their caregivers, specialist physicians and specialist nurses:

1. Conversations took place between pairs of patients with SSc-ILD who discussed their journey and experiences with the disease, and between pairs of specialist physicians and between pairs of specialist nurses who discussed their perspectives on SSc, how they manage patients and any challenges they face in supporting patients. For both patients and HCPs, the focus was on the information sought and exchanged between them and any unmet needs for information. These conversations were spontaneous and free-flowing but facilitated by a professional, experienced qualitative interviewer.
2. A series of mock consultations were conducted between patients with SSc-ILD and specialist physicians. Immediately following the consultation, patients and specialist physicians were interviewed by researchers to capture their thoughts and feelings relating to the consultation and to SSc-ILD more generally. Cognitive language maps were used to evaluate the three most important pieces of information provided and gathered, and what each participant was thinking but did not say. The consultations between patients and physicians were observed but not guided by a moderator. The interviews following the consultation were guided by a professional, experienced qualitative interviewer and conducted in the native language.
3. Caregivers were individually interviewed by a professional, experienced qualitative interviewer in the native language.

Patient, physician and specialist nurse guides for face-to-face interviews were developed for this study (Additional file 1). All conversations, consultations and interviews were audio recorded and transcribed. Patients were under the care of a rheumatologist prior to the study, and consultations were based on an assumed situation where the patient had transferred to a different physician. Patients had differing levels of knowledge about their disease and the possible outcomes.

Procedures followed were in accordance with the Declaration of Helsinki and all participants provided written informed consent before taking part in the study. All study data was held according to European Union data protection laws. The research outline was discussed with an Independent UK Research Ethics Committee who advised that no ethics committee review was required. This report conforms to the Standards for Reporting Qualitative Research guidelines [11].

### Participants

All participants were recruited using researchers based in the respective countries. Patients were recruited by physicians who verified their SSc-ILD diagnosis.

Specialist physicians were consultant-grade rheumatologists or pulmonologists with relevant specialist experience. They were required to be regularly managing patients with SSc-ILD, conducting ≥ 1 consultation every 2 months and seeing ≥ 4 patients with SSc-ILD per year. Additionally, they were required to spend at least 75% of their time directly caring for patients.

Patients with SSc-ILD were diagnosed with lung involvement by a medical specialist using high-resolution computed tomography (HRCT) or lung biopsy. Patients had ILD that limited their ability to conduct moderate or vigorous-intensity physical activity. Patients with a wide range of disease severities and involvement of other organs were included.

None of the physicians or patients were known to one another. Real names or personal identifying information were not provided, and physicians were instructed not to give treatment advice.

Caregivers of the patients with SSc-ILD recruited to this study were invited to participate.

Nurses were specialists in rheumatology, dermatology or pulmonology who had an active role in assessing and managing patients with SSc-ILD for ≥ 2 years. They were also involved in the management of ≥ 1 patient every 6 months (and ≥ 4 per year in total).

### Data analysis

All recordings were transcribed into UK English by specialist medical translation agencies, using a system of forwards and backwards translation to ensure that meaning was retained. Audio transcripts from conversations and interviews were analysed by three different analysts using a technique based on critical discourse analysis for the type of language (words, phrases), intonation, register, pace, etc., used in each setting. Where there was discrepancy, the analysts discussed and agreed on a modified analysis.

## Results

### Observed conversations and interviews

Conversations and interviews with 42 physicians who treat patients with SSc (rheumatologists, pulmonologists, internal medicine specialists, dermatologists), 21 patients with diagnosed SSc-ILD, 16 specialist nurses and five caregivers were recorded (November 2016 to January 2017). The sample size was pre-determined by disease rarity, and patients were selected to represent a range of SSc-ILD severities.

Table 1 shows a summary of the participants.

**Table 1:** Participants included in the analysis, according to country

**Conversations in pairs**	**Physician pairs**	**Specialist nurse pairs**	**Patient pairs**
Germany	3 rheumatologists, 1 pulmonologist	2	4
Italy	3 rheumatologists, 1 pulmonologist	2	4
Spain	3 rheumatologists, 1 pulmonologist, 2 internal medicine	0	2
UK	0	2	4
USA	2 rheumatologists, 1 pulmonologist, 1 dermatologist	2	4
Totals	18	8	18
**In-depth interviews**	**Physicians**	**Patients**	**Caregivers**
Germany	1 rheumatologist, 1 pulmonologist, 1 dermatologist, 1 general practitioner	5	0
Italy	3 rheumatologists, 1 pulmonologist	4	2
Spain	2 pulmonologists	2	1
UK	0	4	1
USA	2 rheumatologists, 2 pulmonologists	4	1
Totals	14	19	4

Patients were aged 34 to 79 years, with a disease duration of 1 to 29 years, and most experienced symptoms involving multiple organ systems including skin, gastrointestinal tract and musculoskeletal. All SSc-ILD was diagnosed under the supervision of a specialist physician by HRCT or lung biopsy.

Physicians treated between 2 and 400 patients with SSc every year and had between 4 and 30 years’ experience in their specialty. All caregivers supported a patient who also took part (one mother, two husbands, one friend and one son).

### Patient/caregiver–physician interactions

In their interactions with physicians, patients focused mainly on giving the physician the information he/she was seeking. Patients often felt too uncomfortable or afraid to ask their physicians questions that were important to them, including asking about their prognosis or future death. Patients sometimes felt intimidated or embarrassed when asking about topics that they felt were ‘out of scope’ for the physician, such as questions about day-to-day management of the symptoms, relationships, family and work. Patients also felt that there was no time during the consultation to ask these questions.

“The doctor said ‘is there any other question that you want’ and I said no, I said I didn’t want to ask that question we all want to ask; she knows, she already knows what I wanted to ask and then we all looked into each other’s eyes and smiled and we left the subject there.” [Patient in Spain]

“Sometimes a couple of things stop you. One is that they’re talking about really serious stuff, like they think your liver is damaged and they don’t know what they are going to do, so we have to consult with three doctors and they’re talking about a serious topic, and you’ve got a question in your thing, ‘Can I fly to Toronto?’ because he’s told me not to get on a plane because I was on immunosuppressants. I want to go on a mini vacation to see my cousin, which is irrelevant.” [Patient in the USA]

Patients and caregivers wanted some certainty and predictability about SSc to enable them to develop a plan for managing the disease following diagnosis. Some patients also wanted reassurance that the physician was in control and had a clear idea of what the patient would need to manage the condition. Other key issues for patients involved work, eating and drinking, exercise, going on holiday, physical appearance, relationships, pregnancy and children, and intimacy and sexuality. The interviews revealed that while patients had variable levels of knowledge and understanding about SSc, caregivers had a poor knowledge of SSc, but both patients and caregivers highlighted that the same questions were of importance to them.

“[On diagnosis] You are left hanging in the air, how does one feel, yes, quite overwhelmed . . .” [Patient in Germany]

“ . . . at the beginning I felt scared because of all the things that they told me [about the effects of the illness].” [Patient in Italy]

“I don’t even know what scleroderma is . . . I don’t even know what the symptoms are, I don’t know why she would have this other than, I don’t know, she doesn’t eat. I don’t know if her muscles just gave away . . .” [Mother of patient in the USA]

“I think the million-dollar question would be to ask them, ‘Is this thing one day going to, you know, get as bad as it can be?’” [Husband of patient in the UK]

Physician language generally was unemotional and matter of fact, full of technical terms but not of imagery or metaphor, which created a barrier to communication. Physicians tended to control the tone and flow of the conversation and thus had a greater ability to emphasise and explore specific parts of the patient experience. This approach resulted in physicians getting targeted information, but the patient left feeling that they were unable to tell their whole story.

When asked, most physicians said that they tried to avoid questions about mortality and prognosis, which were the topics of most importance to patients and caregivers. Physicians’ reasons for doing so included believing it was their duty to give patients some hope and not cause distress, and because they found it difficult to predict what will happen and when with each individual patient. Similarly, physicians often felt it was impossible for them to prescribe a management plan at the time of diagnosis given the unpredictability of the disease. In a few instances, physicians appeared openly negative about the prognosis and did little to see through the disease to the needs of the patient themselves. This caused the patient to ‘close up’.

This led to the patient being left with many unanswered questions, creating feelings of frustration. In addition, it sometimes meant that patients did not volunteer information that might be important for physicians, either because they had no opportunity to do so, because they were embarrassed, or because they felt the physician would not be interested.

“You should just have one of those pains. I have a whole lot, not just the arthritis, the constipation, breathing, you know? I held that back [from the physician], yes. [new pain in hands].” [Patient in the USA]

“‘Do you smoke?’ I said, ‘No,’ and then he didn’t say, ‘Well, have you ever smoked?’ because most people ask, ‘Have you ever smoked?’ The same with the drinking question. He said, ‘Do you drink?’ I said, ‘No,’ but I only haven’t drunk for one year because I was on these medications. Before that I drank, so he didn’t go the next step to ask more questions.” [Patient in the USA]

**Fig. 1:**
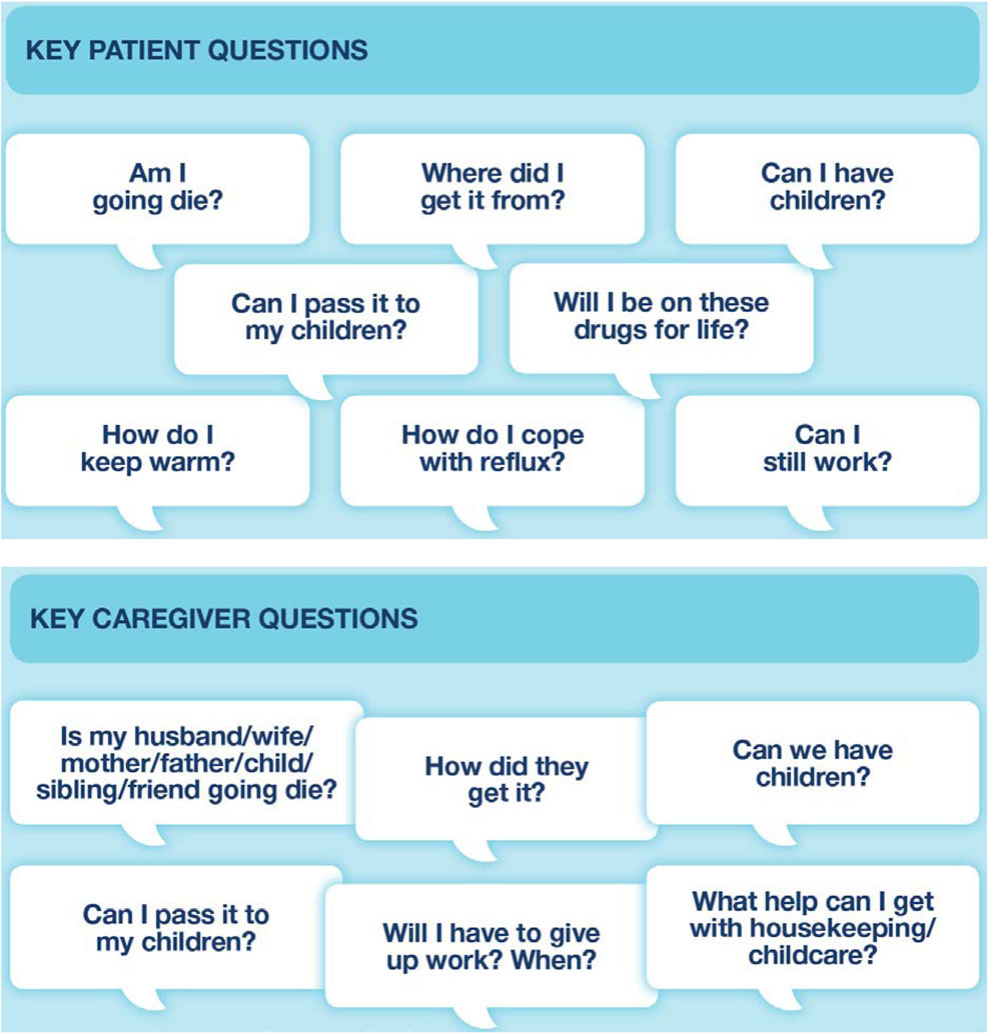
Key patient and caregiver questions

From conversations between patients, patient–physician consultations and patient and caregiver interviews, it was clear unspoken topics were mainly surrounding SSc prognosis, mortality and the impact of SSc on work and family life (Table 2 and Fig. 1).

**Table 2:** Key topics patients with SSc-ILD want to discuss with healthcare professionals that are often omitted from consultations

**Prognosis and mortality**	• The cause of SSc • Death and dying • How they can prevent exacerbations • Wanting to return to their previous life
**Symptom management and wellbeing**	• Nutrition • Keeping warm (Raynaud’s phenomenon) • Coping with reflux • The need for taking drugs for the rest of their life • The effectiveness of treatment
**Family issues**	• Ability to, and safety of, having children, especially if on medication • Heritability of SSc
**Daily life**	• Eating and drinking • Being able to continue working • Practical support with everyday jobs, household chores, etc. • Finances • Exercise • Going on holiday
**Personal issues**	• Physical appearance • Intimacy and sexuality

### Patient/caregiver–nurse interactions

When asked about their interactions with nurses, many patients said that they would feel more comfortable asking a specialist nurse about their concerns, especially questions that they were too embarrassed to ask their physician.

Nurses thought communicating with patients and caregivers who have difficulty accepting the inevitable progression of SSc is particularly difficult, including trying to answer questions about prognosis. This was because of the variability observed in the course of SSc and because they did not have adequate or appropriate information or knowledge. Notably, they often saw this type of question as the physician’s responsibility.

“I think we cannot just spew the same information to all of these different patients because their experience base and their knowledge base are going to be individualised with each individual patient. We have to tailor it so that they can comprehend it.” [Nurse in the USA]

“Diagnosis, progress and prognosis is not for us . . . ” [Nurse in Italy]

“You also have to be careful not to just dismiss everything and end up saying, ‘It’s not so bad, everything will be OK.’ The thing is to find out exactly what the patient’s specific concerns are, and to see if there’s any way you can resolve them.” [Nurse in Germany]

Nurses recognised the differences in patients’ levels of knowledge and understanding about scleroderma, and in their needs for information and support. Some patients need lots of detailed information; others only want the key headlines. However, nurses reported the main challenge with managing patients with SSc-ILD was helping them to cope with the severity of their diagnosis and the emotional burden associated with this. Despite nurses’ acknowledged lack of disease information or knowledge about SSc, they appeared to be the main provider of information and support to patients and caregivers. They appeared to have more time than physicians to take patients through disease information and clarify the messages provided by the physician. During nurse–nurse conversations, it was apparent patients with SSc-ILD talk much more openly with nurses than with physicians, and nurses are generally more concerned with the emotional aspects of the disease. Nurses acknowledged that questions about disease progression, morbidity and mortality were key for patients, but, like physicians, they also found these topics difficult to address.

“It’s kind of like getting a cancer diagnosis. You really don’t want to hear what the doctor has to say right then and there, so then we do schedule them to come back for a follow-up as well, just to, kind of, reiterate what was mentioned and tell them what’s going to go on. We do highly encourage them to bring a family member with them just because, you know, once people hear some sort of a diagnosis, they kind of just turn it off. They don’t want to hear anything else.” [Nurse in the USA]

“. . . the way we nurses talk to a patient is different from the way that the doctor talks to a patient . . . I find that, when I talk to the patients, they are more open with me. You can see that they are a bit more relaxed, whereas when they are with the doctor, they are quite tense, or they are, like, a stuck track. They can’t say anything, they are mesmerised, kind of thing, but when I’m talking to them, they are able to ask questions, or even to open up, even a bit more than they were with the doctor.” [Nurse in the UK]

“I think the medical angle is one thing, but all the other aspects of the care we provide are sometimes much more important than the consultation with the doctor.” [Nurse in Italy]

During nurse–patient interactions, nurses were able to reinforce information provided by physicians. And during nurse–caregiver interactions, nurses were able to better explain information to caregivers around the disease—as patients had a mixed understanding and were not always able to explain to their caregivers what was happening to them. Moreover, nurses were able to recognise the differences between patients’ approaches to their SSc and tailor the level of information provided to each patient. While specialist nurses are in an ideal position to help to break down barriers between patients and HCPs, they often felt they had insufficient disease information or knowledge to provide adequate support and answer questions fully.

### Patient and caregiver perceptions of information sources

Patients and caregivers expressed frustration at the lack of opportunity to obtain clear information, and patients also felt the information that they had seen lacked relevance. For caregivers, this was partially due to their limited access to HCPs, and few caregivers were aware that there were specific support services available to them. These support services might be able to provide the reliable disease-specific information they sought. Most caregivers resorted to the internet to find answers to their questions but were aware when doing so that the quality of internet-based information is not always good. Moreover, they often found information that was incomplete, conflicted with what the physician had told them, or did not seem relevant to their situation.

“I’m not a fan of the internet because it leaves you kind of. .. every website writes something different and it leaves you totally confused and you can get stuck.” [Friend of patient in Germany]

"We started to read about it [SSc] but we are a bit lost with the issue because all we’ve found in the internet ends up increasing our fears instead of providing information.” [Husband of patient in Spain]

## Discussion

It has been suggested that for most patients with SSc-ILD, the specialist physician is the preferred source of information about the disease [12]. There is an expectation from patients that a holistic approach to their wellbeing should be taken, covering all aspects of the disease and its impact [9]. However, our study revealed that questions concerning prognosis and mortality, which are often the most important to patients with SSc-ILD, were commonly overlooked or avoided during discussions between themselves, their caregivers and specialist HCPs. Patients and caregivers often felt uncomfortable asking physicians about SSc-related mortality and prognosis while HCPs found it difficult to provide answers because of the complex and variable nature of SSc and SSc-ILD. Currently, there are no biomarkers or other indicators to predict which patients with early-stage SSc will progress [4], and the only variable that independently predicts mortality and ILD progression is the extent of disease observed on HRCT scans.

This lack of validated, accurate predictors of progression and mortality makes conversations about prognosis in SSc and SSc-ILD difficult: does the physician list all the possible outcomes, with the risk of overwhelming the patient, or shy away from this issue completely and avoid discussing prognosis? Most physicians would welcome a more open and frank discussion with their patients. However, they require clear guidance regarding what patients want to talk about—it is not always obvious what is troubling patients most; for example, from the physician’s perspective, a patient with only minimal skin changes should perhaps focus on more ‘severe’ symptoms, but the patient may feel that this is their primary issue because of concerns about disfigurement [2]. In another example, as women are more often affected by SSc than men, and as onset typically occurs during childbearing years, they may want advice concerning pregnancy [13].

A consequence of patients not feeling able to ask physicians about their concerns was that the unasked questions were then targeted at nurses. They often felt that nurses would be more approachable, and the nurse would also ask them more detailed questions about topics related to day-to-day living, family, relationships and work life. However, nurses may not be as aware of the clinical characteristics and treatment plan for each patient as the treating physician. Our research showed that nurses indeed provided the main source of emotional and practical support for patients, but only those with a specialist interest in SSc are able to provide more specific clinical guidance.

A limitation of the current study, given that it focused on Western populations, is potential generalisability of the findings from this small sample to a broader population. A strength of this study in relation to previous research was that the consultations between patients and physicians were observed but not guided by a moderator. This provided a real insight into the dynamics of the consultation and how the flow of the conversation is directed. Previous research has flagged understanding of SSc and its consequences as well as personal control and emotional representations as the main differences in perceptions between patients and rheumatologists or general practitioners [8]. Studies have also indicated that patients feel physicians are dismissive of their concerns regarding topics such as leisure and social activities, sexuality or parenting [9, 14]. We found that patients mainly provided information that physicians were seeking, and felt intimidated or embarrassed about asking questions related to managing symptoms and how this affects their working life or their relationships.

Although no serious consequences of unanswered questions for patients were identified, it was clear that the questions had the potential to cause unnecessary stress or distress and could possibly impair outcomes in terms of health and wellbeing. By not obtaining all relevant information during the consultation, physicians may develop treatment plans that do not consider the aspects of SSc and its management that are of most importance to the patient. Unanswered questions can be a source of mistrust and anxiety for patients, which can lead to patients withholding information in future consultations, thus creating a vicious circle. Unanswered questions may also drive patients and caregivers to find information, often poor in quality, from other sources. Physicians and nurses both advise patients against searching for information online because of the questionable accuracy of the sources.

Our findings suggest the relationship between patients and physicians is fragile and can be easily damaged by mistrust developed through poor communication. The ‘white coat’ barrier persists; topics of importance to patients and caregivers are often not discussed with physicians. These are generally about the impact of SSc and SSc-ILD on the wider aspects of patients’ and caregivers’ lives; their relationships, family and work and topics around prognosis and mortality are of key importance to patients, caregivers, physicians and nurses, but these are rarely openly discussed. This can create uncertainty and leave patients anxious about the future. Patients and caregivers with unanswered questions unconsciously withhold information about their disease experience, and this has the potential to sway physicians’ choices of the treatment plan. Patients with unanswered questions can experience more stress and may agree to treatment plans that do not align with their personal priorities. By proactively facilitating communications between patients, caregivers, physicians and nurses around perceived difficult topics, information gaps can be minimised and more personalised care delivered. This could lead to better clinical and personal outcomes for patients and enable them to better manage and plan their lives with SSc-ILD; as such, it should be investigated further in future research. Educational resources could also be improved for all participants, closing the knowledge gaps that perpetuate the anxiety around unanswered questions.

## Conclusions

Topics of key importance such as mortality and prognosis are rarely openly discussed within clinical consultations, leaving patients uncertain and anxious about the future. By communicating about difficult but important topics, physicians and nurses could help patients and caregivers manage and plan their lives with SSc. This study shows that a multi-professional team-based communication approach is likely to better address patient needs and priorities. The findings from this research have direct relevance to clinical practice and could be easily implemented without the need for significant additional resources.

## Data Availability

To ensure independent interpretation of clinical study results, Boehringer Ingelheim grants all external authors access to all relevant material, including participant-level clinical study data, and relevant material as needed by them to fulfil their role and obligations as authors under the ICMJE criteria. Furthermore, clinical study documents (e.g. study report, study protocol, statistical analysis plan) and participant clinical study data are available to be shared after publication of the primary manuscript in a peer-reviewed journal and if regulatory activities are complete and other criteria met per the BI Policy on Transparency and Publication of Clinical Study Data: https://trials.boehringer-ingelheim.com/transparency_policy.html. Prior to providing access, documents will be examined, and, if necessary, redacted and the data will be de-identified, to protect the personal data of study participants and personnel, and to respect the boundaries of the informed consent of the study participants. Clinical Study Reports and Related Clinical Documents can be requested via this link: https://trials.boehringer-ingelheim.com/trial_results/clinical_submission_documents.html. All such requests will be governed by a Document Sharing Agreement. Bona fide, qualified scientific and medical researchers may request access to de-identified, analysable participant clinical study data with corresponding documentation describing the structure and content of the datasets. Upon approval, and governed by a Data Sharing Agreement, data are shared in a secured data-access system for a limited period of 1 year, which may be extended upon request. Researchers should use https://clinicalstudydatarequest.com to request access to study data.
